# Operational research on malaria control and elimination: a review of projects published between 2008 and 2013

**DOI:** 10.1186/1475-2875-13-473

**Published:** 2014-12-04

**Authors:** Shui-sen Zhou, Aafje EC Rietveld, Mar Velarde-Rodriguez, Andrew R Ramsay, Shao-sen Zhang, Xiao-nong Zhou, Richard E Cibulskis

**Affiliations:** Global Malaria Programme, World Health Organization, 20 Avenue Appia, CH-1211 Geneva 27, Switzerland; National Institute of Parasitic Diseases, Chinese Center for Disease Control and Prevention, 207 Rui Jin Er Road, Shanghai, 200025 P. R. China; Barcelona Institute for Global Health Hospital Clinic, Universidad de Barcelona, Carrer Rossello 132, 502a, E-08036 Barcelona, Spain; Special Programme for Research and Training in Tropical Diseases (TDR), 20 Avenue Appia, CH-1211 Geneva 27, Switzerland

**Keywords:** Operational research, Malaria, Elimination, Review

## Abstract

A literature review for operational research on malaria control and elimination was conducted using the term 'malaria' and the definition of operational research (OR). A total of 15 886 articles related to malaria were searched between January 2008 and June 2013. Of these, 582 (3.7%) met the definition of operational research. These OR projects had been carried out in 83 different countries. Most OR studies (77%) were implemented in Africa south of the Sahara. Only 5 (1%) of the OR studies were implemented in countries in the pre-elimination or elimination phase. The vast majority of OR projects (92%) were led by international or local research institutions, while projects led by National Malaria Control Programmes (NMCP) accounted for 7.8%. With regards to the topic under investigation, the largest percentage of papers was related to vector control (25%), followed by epidemiology/transmission (16.5%) and treatment (16.3%). Only 19 (3.8%) of the OR projects were related to malaria surveillance. Strengthening the capacity of NMCPs to conduct operational research and publish its findings, and improving linkages between NMCPs and research institutes may aid progress towards malaria elimination and eventual eradication world-wide.

## Background

The past decade has seen tremendous progress in malaria control worldwide. According to the World Malaria Report 2013 [1], 52 of the 97 countries with ongoing malaria transmission are on track to reduce their case incidence rates by 75% by 2015 compared to the year 2000. Four countries have been certified in recent years as having eliminated malaria, and 19 countries are currently implementing pre-elimination or elimination phase. Despite this progress, millions of people who are exposed to malaria risk still do not have access to preventive interventions such as insecticide-treated nets (ITNs); many febrile patients are still treated with antimalarial medicines without a confirmed malaria diagnosis; and malaria surveillance systems are estimated to detect only 14% of all malaria cases worldwide [1]. Operational research (OR) has the potential to improve the outputs and outcomes of malaria programmes [2, 3]. This type of research is useful to assess the feasibility of new interventions in specific settings as well as to identify bottlenecks in malaria control in a particular country [4].

Several authors have highlighted key operational and implementation issues relevant to malaria control programmes, including questions related to malaria in pregnant women [5], challenges faced in scaling up vector control interventions [6], difficulties in implementing case management strategies [7] and weaknesses in national surveillance systems [8]. At the other end of the spectrum, the Research Agenda for Malaria Eradication has identified a number of research priorities that need to be addressed to achieve global eradication of malaria [9]. However, little attention has been given to what OR is being conducted that will help endemic countries progress from malaria control to elimination. To gain a better understanding of the landscape of OR projects in malaria control and elimination, and to support OR agenda setting for malaria elimination, we conducted a systematic review of the malaria literature published over the past five years.

## Methods

### Search strategy, inclusion criteria and data screening

The term 'malaria' was used to search in PubMed all relevant articles published in English between January 2008 and June 2013. The full set of publications thus identified underwent independent screening by two reviewers, using the following definition of OR as inclusion criterion for further analysis: '*the search for knowledge on interventions, strategies, or tools that can enhance the quality, effectiveness, or coverage of programmes in which the research is being done*' [10]. The results of the independent screenings were compared, and when there was disagreement in terms of relevance, the two reviewers met and reached consensus. If consensus could not be reached, a third independent reviewer was consulted to resolve the discrepancy.

### Data extraction

For those publications that met the inclusion criterion, the data was extracted in pre-designed forms. The following information was extracted for each paper: (1) country where the OR project was implemented, (2) institution implementing the project, (3) study methodology, (4) research topic, and (5) relevance to malaria control or elimination.

*Country and region where the project was implemented:* the name of the country where the project was implemented was extracted from each publication. The name of the WHO region to which the country belongs was added. WHO African Region countries were further sub-divided into (a) central, east and southern Africa with high transmission; (b) southern Africa with low transmission; and (c) west Africa. When a project was conducted in more than one endemic country in Africa, it was recorded as ‘multi- African countries’. When a project was conducted in more than one country outside Africa, it was recorded as ‘multi- endemic countries’. If the project was conducted in a malaria-free country the project was recorded as ‘non-endemic country’.

*Institution implementing OR projects:* information about the institution/s involved in the project was gathered. Based on the author’s affiliations, the publications were assigned to the following categories: projects carried out by the ‘National Malaria Control Programme (NMCP)’, by an ‘international research institution’ and/or by a ‘local research institution’.

*Study methodology:* in OR studies a variety of methodologies and study designs can be used, including qualitative, quantitative, experimental and/or non-experimental. For the purpose of this review, the following five methodologies were considered: descriptive (including cross-sectional, if a strong analytic component was present), case–control, cohort (retrospective or prospective), cluster-randomized, and other type of studies (modeling, GIS/GPS/RS and bioassay).

*Programmatic theme and research topic:* based on the objectives of the research project, each publication was recorded as relevant to one of the following thematic areas: Diagnosis, Treatment, Prevention, Vector control, Epidemiology and transmission, Surveillance, Health system (including private sector) and Cost-effectiveness of interventions as a type of OR study. These eight broad areas were chosen for their significance to malaria programmes and operational research. The information regarding the specific research topic investigated in each of the studies was also recorded in the database, and grouped by the eight thematic areas.

*Relevance to malaria control or elimination:* a research project was considered relevant to malaria elimination when at least one of the following criteria was met: (1) the main topic of the publication was regarded as important for malaria elimination, e.g. investigations about the asymptomatic reservoir or prevention of reintroduction; (2) the country where the project was implemented is classified as being in the pre-elimination or elimination phase by WHO [1]; (3) the publication stated the relevance of the project to malaria elimination. All other projects were classified as relevant to malaria control.

### Data analysis

All the information was integrated into an Excel database. A descriptive statistical analysis was conducted for each variable using SPSS 16.0, including the frequency, weight and percentage. The results were sorted by both frequency and percentage for each variable to identify the top ten.

## Results

### Project selection

**Figure 1:**
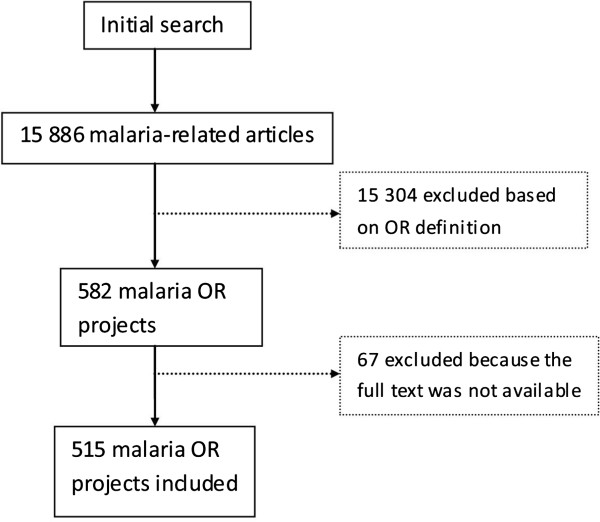
**Selection of malaria OR projects that were included in the analysis.**

15,886 malaria-related publications were identified through our search strategy. Of these, 15,304 did not meet the inclusion criterion and were not considered operational research. Of the remaining 582 OR projects, another 67 publications were excluded because their complete text was not available. For the remaining 515 articles (3% of the total) the complete text was available, and these were included in the analysis (Figure 1).

### Regions/countries where the OR projects have been implemented

**Figure 2:**
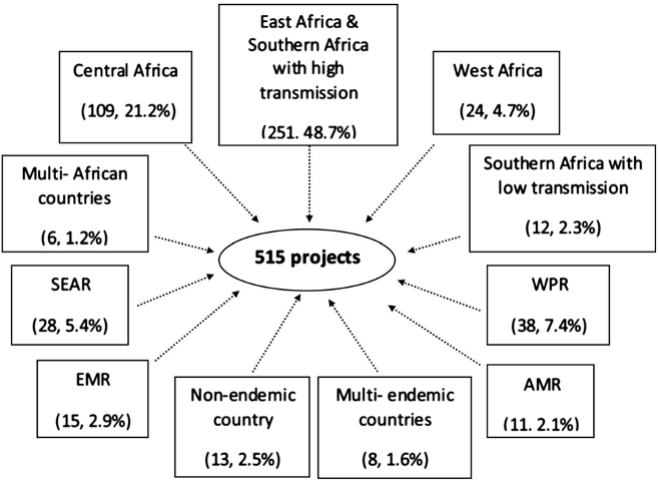
**Regions where OR projects have been implemented, 2008–2013.** AMR: Region of the Americas EMR: Eastern Mediterranean Region. WPR: Western Pacific Region SEAR: South-East Asia Region.

The 515 research projects that met the selection criteria had been implemented in 83 endemic countries around the world. Nearly 77% of the projects were conducted in the WHO African Region, in Africa south of the Sahara. Eighteen percent of the projects were implemented in other WHO regions: the Americas (AMR, 2.1%), Eastern Mediterranean (EMR, 2.9%), South-East Asia (SEAR, 5.4%), and Western Pacific (WPR, 7.4%). None were implemented in the WHO European Region, which aims to eliminate malaria by 2015. In all, 2.5% of projects were conducted in non-endemic countries (related to importation). Although the vast majority of the projects were implemented in only one country, a few projects were carried out in multiple countries (Figure 2).

With regards to the top 12 countries where OR projects have been most frequently implemented (Table 1), United Republic of Tanzania takes the lead with 79 papers, followed by Kenya with 55 papers. Ten of the top 12 countries are located in Africa; only India and the Solomon Islands are outside the African Region. The vast majority of the projects (96%) were conducted in countries in the control phase. Only 5 published OR projects (1%) had been implemented in countries that are classified as being in the pre-elimination or elimination phase [1].

**Table 1:** **Top 12 countries where OR projects were most frequently implemented, 2008-2013**

No.	Country	projects	Percentage
1	Tanzania	79	15.3%
2	Kenya	51	9.9%
3	Nigeria	44	8.5%
4	Uganda	35	6.8%
5	Ethiopia	33	6.4%
6	Ghana	19	3.7%
7	Zambia	17	3.3%
8	Benin	12	2.3%
9	Burkina Faso	12	2.3%
10	Cameroon	9	1.7%
11	India	9	1.7%
12	Solomon Islands	9	1.7%

### Institutions implementing operational research projects

**Figure 3:**
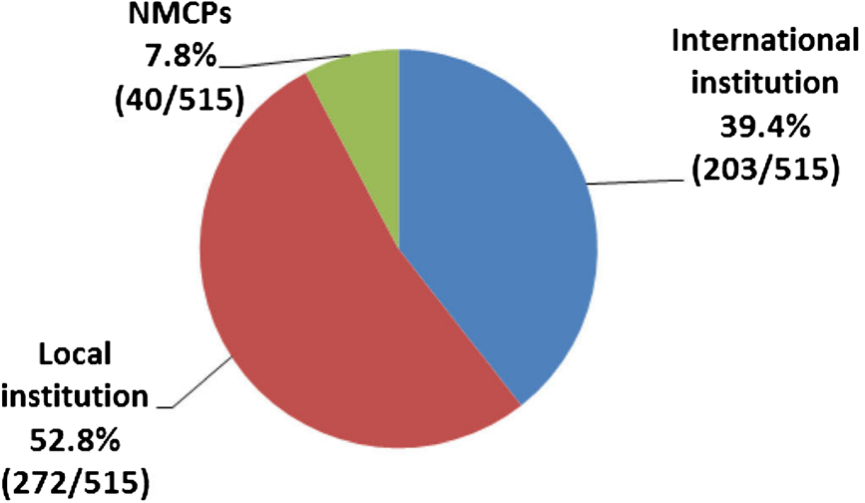
**Type of institution leading the implementation of OR projects, 2008–2013.**

According to this analysis, 92% (475/515) of the OR projects were led by research institutions, both local and international. The remaining 8% (40/515) of OR projects were led by the National Malaria Control Programme (NMCP) (Figure 3). Our study identified 91 international research institutions that have been involved in 304 published OR projects related to malaria in the past five years. Among these, nine universities together with the US Centers for Disease Control and Prevention (CDC/Atlanta) made up the top 10 (in declining order of frequency): London School of Hygiene and Tropical Medicine, Swiss Tropical and Public Health Institute, University of Oxford, Karolinska Institutet, University of Queensland, Johns Hopkins Bloomberg School of Public Health, Liverpool School of Tropical Medicine, Tulane University School of Public Health and Tropical Medicine, and University of California.

### Study methodology

**Figure 4:**
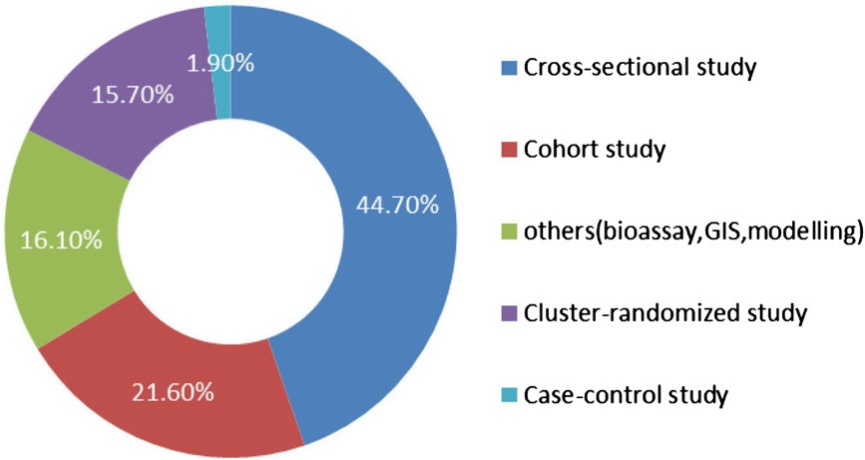
**Study methodologies commonly used in OR studies, 2008–2013.**

Based on the findings, the cross-sectional study was the methodology most commonly (45%) used for malaria OR published between 2008 and 2013. The cohort and cluster randomized studies were also frequently used, accounting for 22% and 16% of the publications respectively. Other types of methodologies such as GIS, modeling and bioassays have also been applied to OR over the past years. However, case–control studies have rarely been used (2%) for malaria OR (Figure 4).

### Research topics

Table 2 lists the thematic areas and specific topics of the 515 OR studies published between 2008 and 2013. A large number of OR study projects were classified as relevant to vector control (25%, 129/515); followed by epidemiology and transmission (16.5%, 85/515); and treatment (84/515, 16%). Only a small number of projects examined issues concerning malaria surveillance (19/515, 4%).

**Table 2:** **Thematic areas and specific topics**

Programmatic themes	Number	Percent	Research topics	Number	Percent
Diagnosis	47	9.1%	RDT	25	4.9%
			Parasitologically-confirmed diagnosis	9	1.7%
			Diagnostics comparison	10	1.9%
			Perception & adherence to guidelines	2	0.4%
			QA/EQA	1	0.2%
Treatment	84	16.3%	G6PD	3	0.6%
			Anti-relapse and Primaquine	6	1.2%
			Accessibility to treatment	10	1.9%
			Adherence to treatment	15	2.9%
			Home management	18	3.5%
			Efficacy and resistance to antimalarial	5	1.0%
			Treatment perception & behaviour	27	5.2%
Prevention	52	10.1%	Community perception & practice	14	2.7%
			Prevention & IPTp	27	5.2%
			Prevention & IPTi	5	1.0%
			Prevention & IPTc	5	1.0%
			Chemoprophylaxis	1	0.2%
Vector control	129	25.0%	Perception & acceptability of IRS	5	1.0%
			Perception & acceptability of bednets	32	6.2%
			Environment management	5	1.0%
			Mosquito sampling methods	5	1.0%
			ITN vs IRS and combination	5	1.0%
			Ownership & utilization of bednets	40	7.8%
			Bionomics of vector & efficacy of insecticide	21	4.1%
			Larvae & larviciding	4	0.8%
			Impact of anti-vector intervention	5	1.0%
			Outdoor-biting	4	0.8%
			Sustain or phase out of vector intervention	1	0.2%
			IVM	1	0.2%
			Geographical Reconnaissance (GR)	1	0.2%
Epidemiology & transmission	85	16.5%	Infection reservoir-asymptomatic, submicroscopic, gametocyte carriers	15	2.9%
			Risk factor & Prevalence	22	4.3%
			Hotspot & foci	7	1.4%
			Importation	13	2.5%
			Elimination strategy	2	0.4%
			Reintroduction	4	0.8%
			Risk & distribution mapping/model	22	4.3%
Surveillance	19	3.8%	Reporting & quality of data	6	1.2%
			Mobile phone-based case detection	3	0.6%
			ACD vs PCD	8	1.6%
			Surveillance as intervention	1	0.2%
			Early warning & response	1	0.2%
Health system	73	14.2%	Health worker's perception & practice	27	5.2%
			Availability & use of service	7	1.4%
			Health facility performance	19	3.7%
			Provision of service by private sectors	6	1.2%
			Perception & adherence to guidelines of private sectors	5	1.0%
			Subsidy policy to private sectors	8	1.6%
			Malaria reporting by private sectors	1	0.2%
Cost-effectiveness	26	5.0%		26	5.0%
**Total**	515	**100.0%**		**515**	100.0%

With regards to the specific research topics, 50 different research topics were identified. The most frequently investigated topics included questions about RDT-based diagnosis; treatment perception and behavior; prevention and IPTp; perception, acceptability, ownership and utilization of bed nets; health worker’s perception and practice. Each of these issues accounted for 5% of the total number of projects published between 2008 and 2013. Questions of direct relevance to malaria elimination have been less studied. For instance, only one project each studied quality assurance of microscopic diagnosis, phasing out of vector interventions, geographical reconnaissance, and surveillance as an intervention.

### Relevance to malaria control and elimination

Ninety-three percent (478/515) of the projects were classified as being relevant to malaria control, and 7% (37/515) were relevant to malaria elimination. 27 of the latter 37 studies were included because the main topic of the publication was regarded as important for malaria elimination (e.g., asymptomatic infection and gametocyte carriers, malaria re-introduction, community participation in malaria elimination, hotspots and foci, geographical reconnaissance of target populations in malaria elimination zones, case-based surveillance, optimization of strategies for malaria elimination, sustain or phase out of interventions, reactive case detection for malaria elimination); four because the country where the project was implemented is classified as being in the pre-elimination or elimination phase by WHO; and six because the publication stated the relevance of the project to malaria elimination.

## Discussion

The results of this literature review show that although operational research only represents a small percentage of the malaria research papers published between 2008 and 2013, these studies have been carried out in a large number of malaria endemic countries. The malaria OR studies examined questions relevant to a broad range of thematic areas including diagnosis, treatment, prevention, vector control, surveillance, epidemiology and transmission, health systems and cost-effectiveness.

However, it seems that a majority of the OR projects carried out over the past years have focused on countries with high burden, especially in Africa south of the Sahara. Only a small number of projects were conducted in low transmission settings or countries approaching malaria elimination. Furthermore, only a few of these projects focused on topics and activities relevant to malaria elimination, including hot spots [11–13], reactive case detection [14, 15], asymptomatic parasite carriers [16, 17], mobile phone-based surveillance [18–21] and the use of single low-dose primaquine [22]. There remain many research questions that are critical to move forward with malaria elimination [23].

The results of the present analysis show an extensive and active involvement of funding partners as well as research institutes in malaria OR, and a limited involvement of NMCPs. Since the NMCPs have comprehensive knowledge of the malaria situation in their countries and are end-responsible for the implementation of control and elimination activities, closer engagement with NMCPs could benefit the local relevance of OR that is carried out in malaria-endemic countries, and facilitate the uptake of research findings into policy and programme activities. To achieve this, funding partners could support NMCPs more directly, and encourage write-up and publication of NMCP-led OR studies in peer-reviewed journals.

Zachariah *et al.* listed the study methodologies most commonly used in OR as: descriptive (cross-sectional, if a strong analytic component is also present); case–control; and retrospective or prospective cohort analysis, and noted that basic research and randomized controlled trials are not used in operational studies [2]. In this review, the authors tried to classify all OR projects according to these three main types of study methodologies. However, it was found that a number of malaria OR projects employed other methods such as cluster-randomized study, GIS, bioassay and modelling to investigate the bottlenecks encountered during the implementation of programme activities.

Based on the findings, cross-sectional design was the methodology mostly frequently employed and case–control design (1.9%) was the least employed in past years. Since case control studies are more appropriate to investigate diseases that occur at a low incidence, it follows that they should be more frequently used to study malaria in low-transmission settings.

This review has a number of limitations. First of all, the literature search only included articles published in PubMed. While studies carried out by research institutions are frequently published in PubMed, we recognize that OR projects undertaken by the national malaria programmes may not always be published or reflected in PubMed. Moreover, only publications published in English were reviewed and a high number of OR projects may be published in local languages. In future literature reviews on malaria operational research, it will be important to include additional databases and other languages.
